# Distribution and Quantification of Choroidal Macrophages in Human Eyes With Age-Related Macular Degeneration

**DOI:** 10.1167/iovs.16-20049

**Published:** 2016-11

**Authors:** D. Scott McLeod, Imran Bhutto, Malia M. Edwards, Rachel E. Silver, Johanna M. Seddon, Gerard A. Lutty

**Affiliations:** 1 Wilmer Ophthalmological Institute, Johns Hopkins Hospital, Baltimore, Maryland; 2 Ophthalmic Epidemiology and Genetics Service, New England Eye Center, Tufts Medical Center, Boston, Massachusetts; 3 Department of Ophthalmology, Tufts University School of Medicine, Boston, Massachusetts

**Keywords:** age-related macular degeneration, choriocapillaris, choroidal neovascularization, choroidal vasculature, macrophages

## Abstract

**Purpose:**

Increasing evidence suggests a role for macrophages in the pathogenesis of age-related macular degeneration (AMD). This study examined choroidal macrophages and their activation in postmortem eyes from subjects with and without AMD.

**Methods:**

Choroids were incubated with anti-ionized calcium-binding adapter molecule 1 (anti-IBA1) to label macrophages, anti-human leukocyte antigen-antigen D-related (anti-HLA-DR) as a macrophage activation marker, and *Ulex europaeus* agglutinin lectin to label blood vessels. Whole mounts were imaged using confocal microscopy. IBA1- and HLA-DR–positive (activated) cells were counted in submacula, paramacula, and nonmacula, and cell volume and sphericity were determined using computer-assisted image analysis.

**Results:**

In aged control eyes, the mean number of submacular IBA1^+^ and HLA-DR^+^ macrophages was 433/mm^2^ and 152/mm^2^, respectively. In early AMD eyes, there was a significant increase in IBA1^+^ and HLA-DR^+^ cells in submacula compared to those in controls (*P* = 0.0015 and *P* = 0.008, respectively). In eyes with neovascular AMD, there were significantly more HLA-DR^+^ cells associated with submacular choroidal neovascularization (*P* = 0.001). Mean cell volume was significantly lower (*P ≤* 0.02), and sphericity was significantly higher (*P ≤* 0.005) in all AMD groups compared to controls.

**Conclusions:**

The average number of IBA1^+^ macrophages in submacular and paramacular choroid was significantly higher in early/intermediate AMD compared to that in aged controls. HLA-DR^+^ submacular macrophages were significantly increased in all stages of AMD, and they were significantly more round and smaller in size in the submacular AMD choroid, suggesting their activation. These findings support the concept that AMD is an inflammatory disease.

Age-related macular degeneration (AMD) is the leading cause of severe vision loss in patients over 50 years of age in industrialized countries.^1^ Despite its high prevalence, the cause of AMD remains largely unknown. Clinically and histologically, AMD is generally classified as nonexudative or dry AMD, of which geographic atrophy (GA) is the severe form, and exudative or neovascular AMD. Dry AMD progresses more slowly and is characterized by drusen, geographic or focal atrophy of the retinal pigment epithelium (RPE), and photoreceptor dysfunction and degeneration. The advanced exudative form, neovascular AMD, can develop after early and intermediate dry AMD.^2^ The key feature of the advanced subtype is choroidal neovascularization (CNV), the growth of new blood vessels from the choroid into the region underlying the RPE or extending into the subretinal space.

Emerging evidence supports the association between chronic inflammation and AMD. Penfold et al.^3^ described inflammatory cells (macrophages, lymphocytes, and mast cells) in human AMD choroids. More recent findings suggest a role for immunologic processes in AMD pathogenesis, including recruitment of macrophages,^4^ involvement of systemic inflammatory processes,^5–7^ complement activation,^8–12^ and microglial activation and accumulation.^13^ Historically, macrophages have been observed at sites of CNV in patients with AMD,^14,15^ and CD68^+^ macrophages were detected in rapidly progressive fibrovascular AMD membranes.^16^ Grossniklaus et al.^17^ evaluated human CNV and observed that macrophages express proangiogenic vascular endothelial growth factor (VEGF) and suggested that they directly promote CNV.^18^

Macrophage populations are simplistically divided into phenotypes M1 and M2. Phenotype M1 is proinflammatory and causes tissue injury, whereas M2 phenotype is pro-angiogenic and promotes tissue repair.^19^ In the laser-induced mouse CNV model with scar, M2 macrophages were recruited to the site of laser injury^20,21^; however, M1 macrophages were identified in the subretinal space when mice were immunized with carboxyethylpyrrole), an immune response-initiated model of AMD.^22,23^ Determination of M1 and M2 macrophages in a human is not as straightforward as it is in mouse. In the present study, we chose to localize all macrophages with the monocyte marker ionized calcium-binding adapter molecule 1 (IBA1) and determine if they were activated using an antibody to human leukocyte antigen-antigen D-related (HLA-DR), a subunit of major histocompatibility complex (MHC) class II.

The present study aimed to quantify the number, distribution, and activation of choroidal macrophages in choroidal whole mounts from aged control eyes and in eyes from patients with clinically documented early and intermediate AMD, as well as two advanced subtypes, neovascular AMD and GA. The presence of macrophages was related to changes in choriocapillaris (CC) and CNV.

## Materials and Methods

### Donor Eyes

Human donor eyes were obtained from 16 subjects (Table). All tissue was obtained within 10 to 35 hours of death. All tissue were used in accordance with the Declaration of Helsinki and with approval of the institutional review boards at Johns Hopkins University School of Medicine and Tufts Medical Center. All donors were Caucasian. The Table summarizes the characteristics of each donor subject. The diagnosis and severity of AMD were determined by clinical examination (JMS), using the Clinical Age-Related Maculopathy Staging (CARMS) system,^24^ and by postmortem gross examination of posterior eyecup, using transmitted and reflected illumination with a dissecting microscope (Stemi 2000; Carl Zeiss, Inc., Thornwood, NY, USA). During gross examination, eyes were classified according to severity of disease: early AMD, defined as soft indistinct drusen with or without pigmentary changes (CARMS grade 2 [*n* = 2]); intermediate AMD, or soft distinct drusen with pigmentary changes (CARMS grade 3 [*n* = 3]); and late (end-stage) AMD (*n* = 8). Late AMD was classified as dry AMD with GA (characterized by thick basal laminar deposits and distinct areas of RPE loss [CARMS grade 4 {*n* = 5}]) or neovascular disease with CNV (CARMS grade 5 [*n* = 3]). Two eyes with neovascular disease were treated with anti-VEGF injections, and one eye was treated with laser therapy. Subjects with a history of diabetes mellitus were excluded from the study.

**Table:**
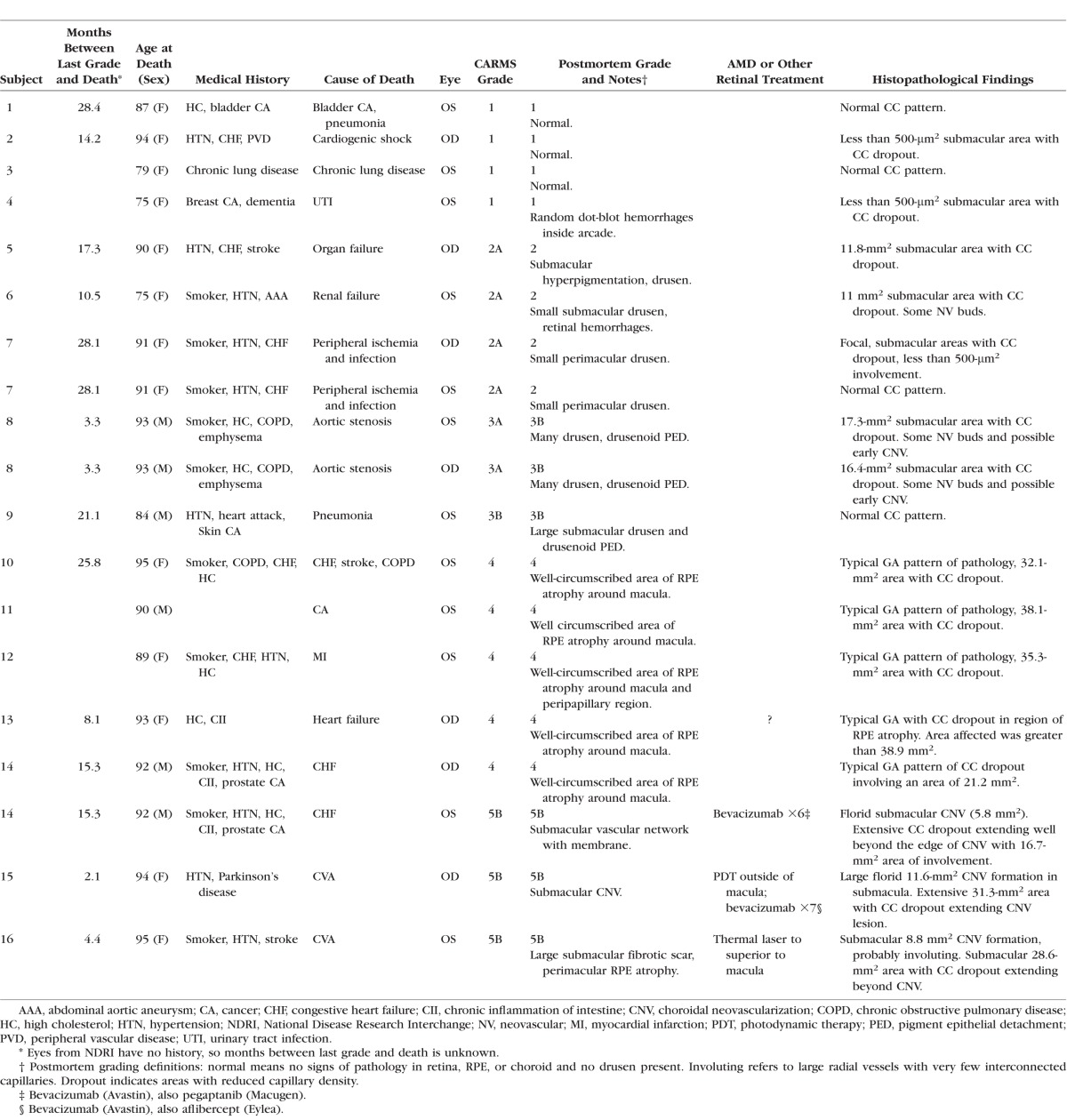
Clinical and Histopathologic Data for Ocular Tissue From Aged Controls and Subjects With Early, Intermediate, and Advanced Stages of AMD

### Tissue Preparation

After removing the anterior segments, we grossly examined the posterior eyecups. Retinal pigment epithelium were removed and choroids excised and fixed as published previously.^25^ Vitreous was removed, and retinas were excised from the RPE/choroid. Gross digital images of eye cups and choroids with and without RPE were captured.

### Whole-Mount Immunohistochemistry

Choroids were washed twice in 0.1 M cacodylate buffer at 4°C and then in Tris-buffered saline (TBS) with 0.1% Triton X-100 (TBS-T) for 10 minutes. After being washed, choroids were incubated with 5% normal goat serum in TBS-T with 1% bovine serum albumin overnight at 4°C. Tissues were then washed with TBS-T and incubated with rabbit anti-IBA1 (1:500 dilution; Wako Chemicals USA, Inc., Richmond, VA, USA) and mouse anti HLA-DR (1:200 dilution; Dako North America, Inc., Carpinteria, CA, USA) for 72 hours at 4°C. After tissues were washed, they were incubated with secondary antibodies (1:200 dilution) for 48 hours at 4°C of goat anti-mouse-Cy3 (Jackson ImmunoResearch, West Grove, PA, USA); goat-anti rabbit- Alexafluor647 (A647; Invitrogen, Carlsbad, CA, USA); and *Ulex europaeus* agglutinin (UEA) lectin/fluorescein isothiocyanate (FITC)-conjugated (1:100 dilution; Sigma-Aldrich Corp., St. Louis, MO, USA). Tissues were washed in TBS and then imaged with a model LSM 710 confocal microscope (Carl Zeiss Microscopy, LLC, Thornwood, NY, USA) at 488-nm, 561-nm, and 633-nm excitation (FITC: Cy3, A647 staining, respectively).

### Image Acquisition

An area from the posterior pole region approximately 8 × 10 mm^2^ was excised from each choroid. This tissue included the region just nasal to the optic nerve opening, beyond the inferior and superior vascular arcades of retina, and several millimeters temporal to the macula (Supplementary Fig. S1). The tissue was flat mounted and imaged at 5×, 10×, and 20× magnification with Bruch's membrane nearest to the objective. The submacular region was centered in the oculars and 5 × 7 tiled overlapping fields (10% overlap) at 2048 × 2048 pixel resolution were collected as *z* stacks using Zen software (2010; Carl Zeiss, Inc.). Laser power, pinhole, gain, and other capture parameters were saved and used for imaging all choroids under identical conditions. The inner boundary of the *z* stack was set at the focus level just above the CC, and the outer limit of the *z* stack was set where IBA-1-labeled cells disappeared in the tissues. For volume renderings, optimized capture parameters were used with a 40× oil immersion objective to obtain single nontiled fields in the submacular region.

### Image Analysis for Cell Counts

Maximum intensity projection 5× stitched images were exported from Zen software as full-resolution tagged image file format (TIFF) images and opened using available software (Photoshop CS6; Adobe Systems, Inc., San Jose, CA). Three 1204 × 1204 pixel dimension selections (equivalent to1 mm^2^) were randomly made of regions in submacular, paramacular, and nonmacular choroid (Supplementary Fig. S1) and pasted into new image documents. Each image was adjusted using the channel mixer function in Photoshop to segregate the different labels (blue for UEA labeled blood vessels, green for IBA1 labeled macrophages and red for HLA-DR stained macrophages). Each channel was copied and saved as separate TIFF images. Levels and thresholding were then adjusted and saved for analysis (Supplementary Fig. S2) using ImageJ software (http://imagej.nih.gov/ij/; provided in the public domain by the National Institutes of Health, Bethesda, MD, USA). These adjustments were predetermined to give the best results for automated counts and used consistently throughout the study. The images were converted to binary, noise reduction was applied, and the analyze-particle function was used to perform cell counts. The cutoff used for the pixel dimension of the particles to count was 50 to infinity. Manual hand counts were done initially by three independent reviewers to verify the accuracy of counts in ImageJ software.

### Volumetric and Sphericity Measurements

Volumetric and sphericity measurements were performed in the submacular region to analyze changes in shape and size of choroidal macrophages. Briefly, optimized 40× magnification *z* stacks were opened in Surpass View of Imaris version 8.1 software (Bitplane USA, Concord, MA, USA) and surfaces created for the IBA1 channel, using background subtraction. Voxel number filtering was applied to remove any nonspecific particles in the choroidal stroma and surfaces created. Cells touching others or ones that had only a portion of the cell body in the field were eliminated from the volume rendering. Volume, area, and sphericity statistics were exported for analysis, and snapshots were saved of the volume renderings.

### Statistical Analysis

Student's *t*-test for two independent samples with unequal variances assumed compared eyes with early and intermediate AMD, GA, and neovascular AMD to aged control eyes. One-way ANOVA analysis with a Tukey-Kramer post hoc test was performed to compare eyes from the three AMD groups in an exploratory analysis. Data are means ± SD. A *P* value less than 0.05 was considered statistically significant for all comparisons.

## Results

### Macrophage Counts and Morphology

**Figure 1:**
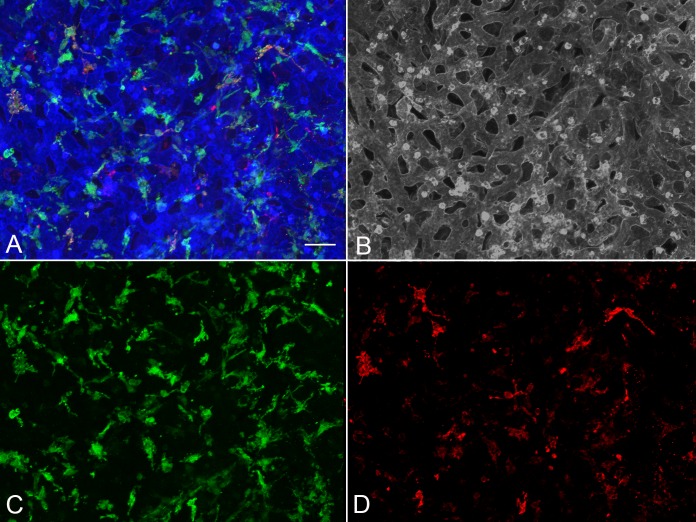
Higher magnification image of a submacular field in the aged control choroid in Supplementary Figure S3 showing a homogeneous pattern of choriocapillaris with broad lumen and freely interconnecting channels (*blue* [**A**]; *gray* [**B**]). IBA1-labeled macrophages (*green*) were generally ramified, with several thick limbs, bearing short fine processes. HLA-DR^+^ macrophages (*red*) were much fewer in number than IBA1^+^ cells. (**A**) Merged channels; (**B**) desaturated UEA channel; (**C**) IBA1 channel; (**D**) HLA-DR channel. *Scale bar*: 25 μm.

**Figure 2:**
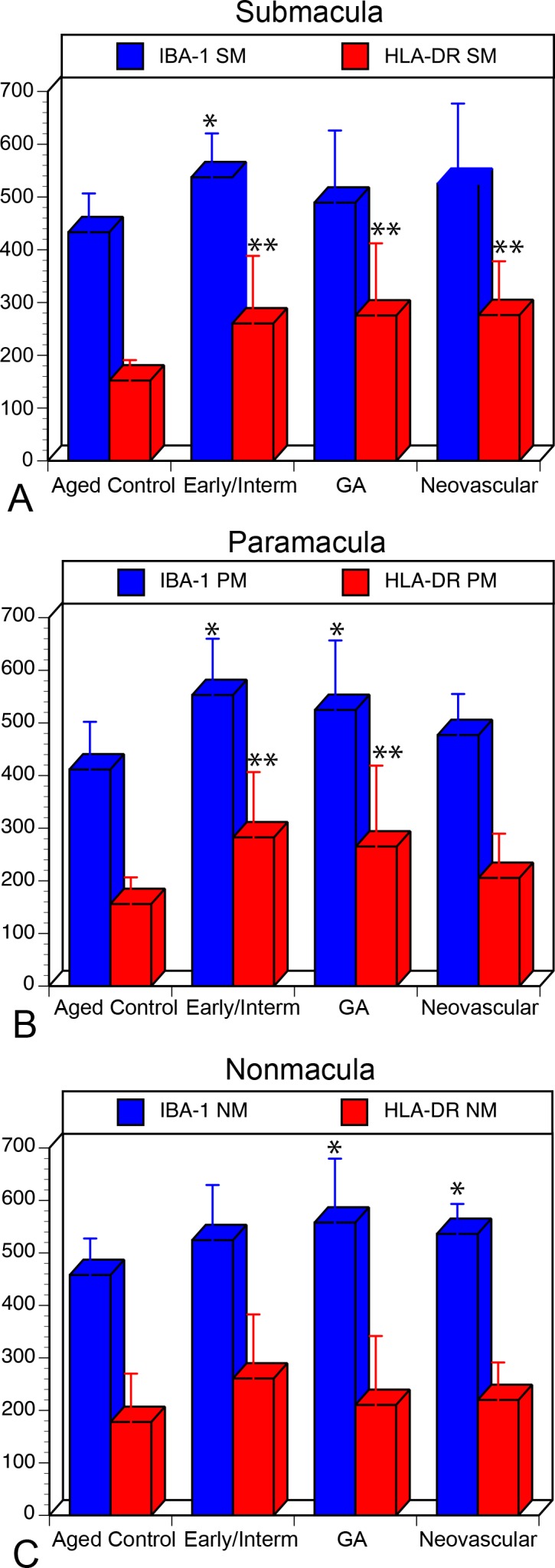
Graphs showing cell counts/mm^2^ in three regions of choroid. (**A**) Submacula; (**B**) paramacula; (**C**) nonmacular, analyzed in aged control eyes, early/intermediate AMD eyes (CARM grades 2/3), GA eyes (CARMS grade 4), and neovascular AMD eyes (CARMS grade 5). There was a significant increase in HLA-DR^+^ macrophages (r*ed*) in the submacula of all AMD groups compared to those in controls. Additionally, there was a significant increase in the number of IBA1^+^ macrophages (*blue*) in early AMD in submacula and paramacular choroid and in nonmacular region in GA and neovascular AMD (*P < 0.05; **P < 0.001).

**Figure 3:**
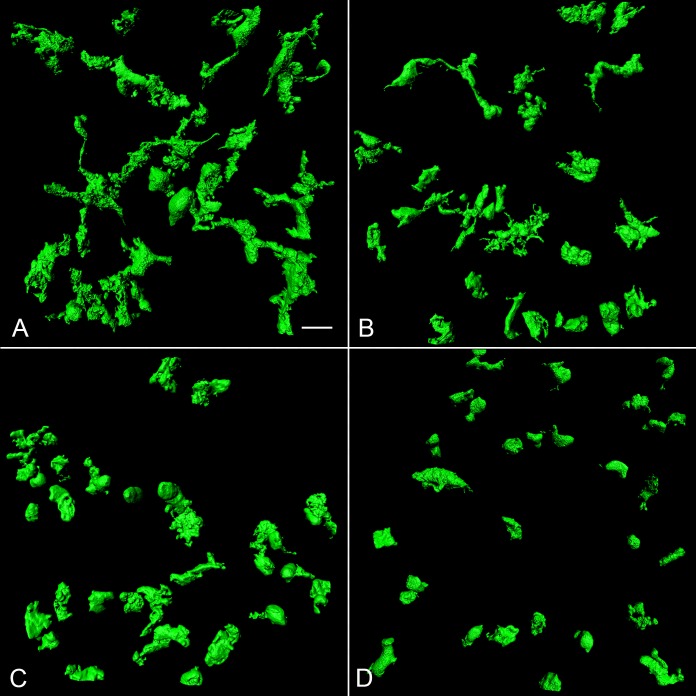
Representative volume renderings of IBA1^+^ macrophages in the submacular choroid of an aged control eye (**A**), early AMD eye (**B**), an eye with neovascular AMD (**C**), and in a subject with GA eye (**D**). Macrophages in the aged control have a large cell volume and a ramified cellular morphology. In eyes with early AMD, macrophages have fewer processes and reduced cell volumes. In advanced AMD (neovascular AMD and GA), macrophages have very few processes, are more rounded, and are much smaller in size. *Scale bar*: 10 μm.

**Figure 4:**
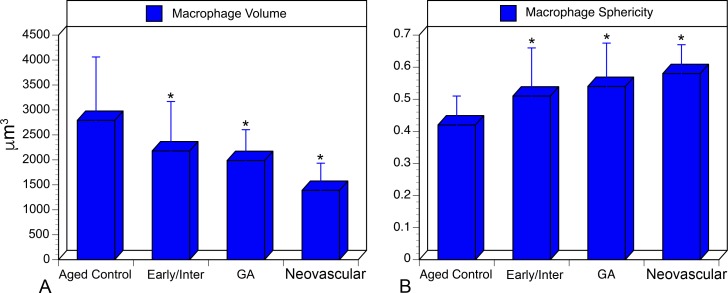
(**A**) Cell volume of submacular choroidal macrophages in aged control eyes, eyes with early/intermediate AMD, GA, and neovascular. There was a statistically significant decrease in cell volume in a comparison between control subjects and each AMD group (*P = 0.03; **P < 0.0009). (**B**) Sphericity of submacular choroidal macrophages in aged control eyes, eyes with early/intermediate AMD, eyes with neovascular AMD, and GA eyes. There was a statistically significant decrease in cell sphericity in a comparison between control subjects and AMD group (**P < 0.005).

Analysis of aged control eyes revealed a homogeneous distribution of CC and IBA1^+^ choroidal macrophages throughout the posterior pole (Supplementary Fig. S3). When viewed at higher magnification, it is apparent that HLA-DR labeling in controls is often only in a portion of the IBA1^+^ cells (Fig. 1). IBA1^+^ cells and HLA-DR^+^ macrophage counts in submacula, paramacula, and nonmacula are shown in Figure 2. Counts from nasal regions of choroid showed similar numbers (data not shown). Macrophages expressing HLA-DR were also uniformly distributed in aged control eyes; however, their numbers were significantly lower (Fig. 2). Examined at higher magnification, aged control choroidal macrophages were generally ramified, with several thick limbs, bearing short, fine processes (Figs. 1, 3). Most of the macrophages were positioned just externally or posteriorly to the CC, and their processes were often in contact with the outer abluminal wall of the capillaries (Supplementary Movie S1). Macrophage volume in the submacular region of aged control eyes was 2791.4 ± 1270.6 μm^3^ (Fig. 4A), and their sphericity was 0.42 ± 0.9 (Fig. 4B).

**Figure 5:**
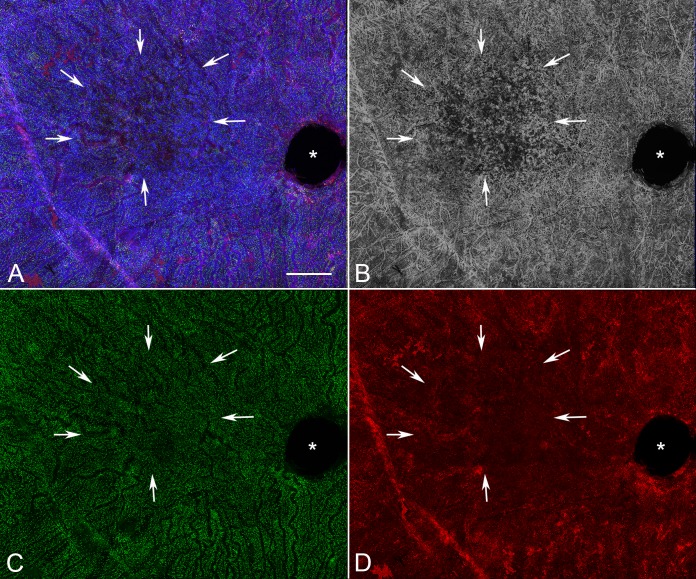
Low-magnification view of the posterior pole region of the choroid from an early AMD subject (subject 6) showing a localized 11-mm^2^ area of CC dropout in the submacular region (*arrows*). At this magnification, there was no obvious increase in either IBA1^+^ (**C**) or HLA-DR^+^ (**D**) macrophages in the submacular choroid. (**A**) Merged channels; (**B**) desaturated UEA channel; (**C**) IBA1 channel; (**D**) HLA-DR channel. *Optic nerve. *Scale bar:* 1 mm.

**Figure 6:**
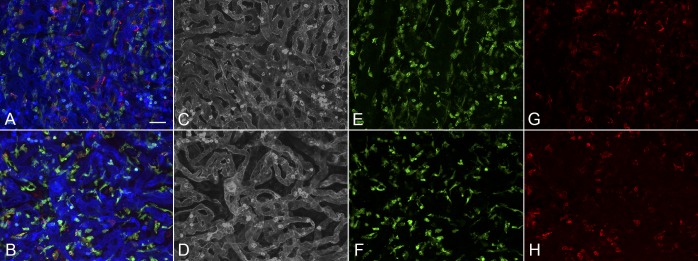
Higher magnification images of the paramacular (**A**, **C**, **E**, **G**) and submacular field (**B**, **D**, **F**, **H**) of choroid in the early AMD subject (subject 6) shown in Figure 6 demonstrates a homogeneous pattern of choriocapillaris with broad lumen and freely interconnecting channels in the paramacular region (**A**, **C**) and an abbreviated pattern with loss of interconnecting capillaries in the submacular region (**B**, **D**). IBA1-labeled macrophages are somewhat more ramified in paramacular choroid (**E**) than those in the submacula (**F**). HLA-DR^+^ macrophages were more numerous in both of these regions (**G**, **H**) compared to aged controls. (**B**) Merged channels; (**C**, **D**) desaturated UEA channel; (**E**, **F**) IBA1 channel; (**G**, **H**) HLA-DR channel. *Scale bar:* 25 μm.

Eyes with early/intermediate AMD showed CC dropout in submacula ranging in area of involvement from less than 500 μm^2^ to as much as 11.8 mm^2^. These eyes generally had a homogeneous distribution of IBA1^+^ macrophages throughout the posterior pole region that was similar to that of controls (Fig. 5). The number of IBA1^+^ cells/mm^2^ was significantly elevated in submacula and paramacula (Fig. 2). The mean numbers of both IBA1^+^ and HLA-DR^+^ cells/mm^2^ were significantly higher in the submacula (*P* = 0.001 and *P* = 0.008, respectively) and paramacula (*P* = 0.0007 and *P* = 0.002, respectively) of early/intermediate AMD eyes than in those of aged controls. In early/intermediate AMD, submacular macrophages appeared significantly more round (*P* = 0.005) and ramified and significantly smaller in size (*P* = 0.03) than those in aged controls, especially where early CC dropout was apparent (Figs. 3, 6)

**Figure 7:**
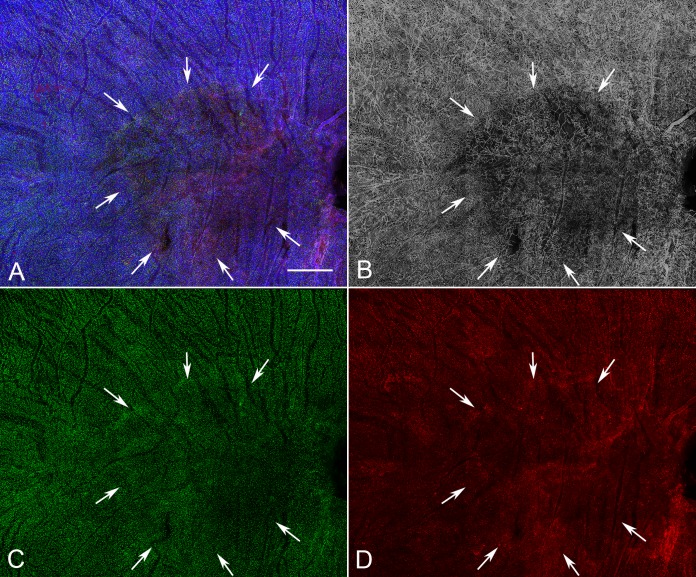
Low-magnification image of the posterior pole region of choroid from a subject with GA (subject 14, OD) showing a well-demarcated area of choriocapillaris attenuation in the region corresponding to RPE atrophy (*arrows*). There appear to be no significant differences in the number of IBA1^+^ macrophages (**F**) in the atrophic, border, or nonatrophic regions of choroid. (**A**) Merged channels; (**B**) desaturated UEA channel; (**C**) IBA1 channel; (**D**) HLA-DR channel. *Scale bar:* 1 mm.

**Figure 8:**
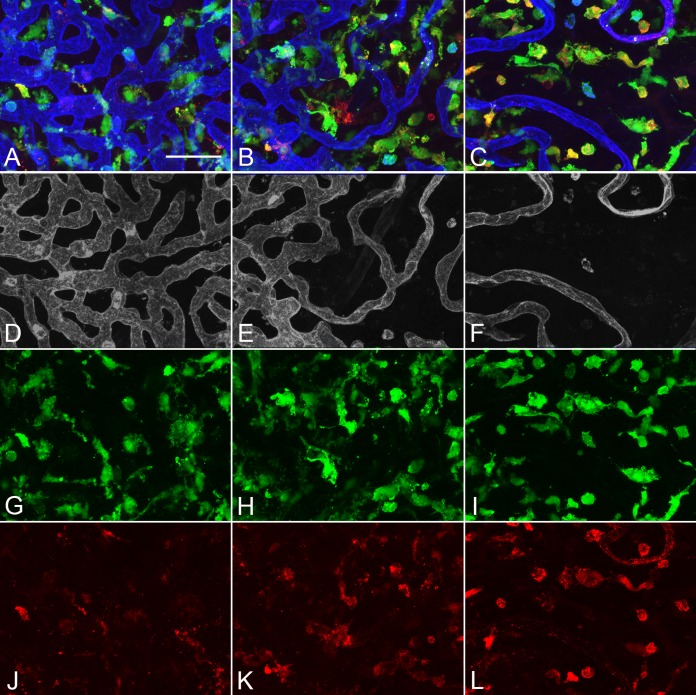
Higher magnification images of the nonmacular, nonatrophic region (**A**, **D**, **G**, **J**), a paramacular/border region to atrophy (**B**, **E**, **H**, **K**), and submacular atrophic region (**C**, **F**, **I**, **L**) in the GA subject shown in Figure 7 (subject 14, OD) demonstrates normal choriocapillaris pattern in the nonmacular region (**D**), an attenuated choriocapillaris at the border of RPE atrophy (**E**), and a severely attenuated choriocapillaris in the region of RPE atrophy (**F**). IBA1^+^ macrophages were more ramified in nonmacular choroid (**G**) then they were at the border of atrophy (**H**) or in the area of atrophy (**I**). There were fewer HLA-DR^+^ macrophages in the nonmacula (**J**) than at the border (**K**) and in the atrophic region (**L**). (**A**–**C**) Merged channels; (**D**–**F**) desaturated UEA channel; (**G**–**I**) IBA1 channel; (**J–L**) HLA-DR channel. *Scale bar:* 50 μm.

There was severe CC dropout in the submacular region corresponding to the area of RPE atrophy in eyes with GA (Figure 7). Higher magnification of the *z* stacks suggested that the macrophages had moved anterior in the choroid, occupying space where CC segments once had been (Supplementary Movie S2). These macrophages had fewer processes and appeared more round. There were significantly more IBA1^+^ cells in the paramacula and nonmacula (*P* = 0.01 for both regions) (Fig. 2). The mean number of IBA1^+^ cells was significantly higher in paramacula and nonmacula than in controls (Fig. 2); however, there were no differences in the submacula. The mean number of HLA-DR^+^ macrophages were significantly higher in the submacular and paramacular choroid of GA eyes than in controls (*P* = 0.006 and *P* = 0.026, respectively). Macrophage volume in eyes with GA was 1986.7 ± 617.9 μm^3^, and sphericity was 0.54 ± 0.13 (*P* = 0.0009 and *P* < 0.00001, respectively). Macrophages were significantly smaller and more rounded in GA eyes than those observed in aged control eyes**.** In paramacular (near the border of RPE atrophy) and nonmacular (nonatrophic) regions of GA choroid, macrophages had a more ramified morphology (Fig. 8).

**Figure 9:**
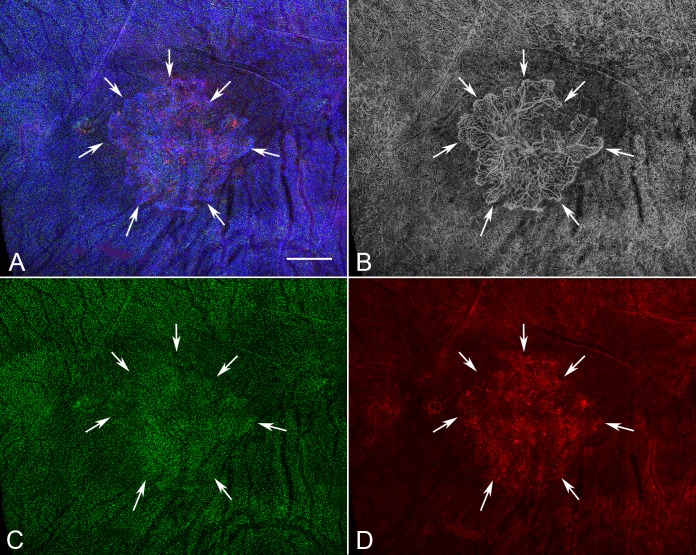
Low-magnification image of the posterior pole region of choroid from a subject (subject 14, OS) with neovascular AMD showing a CNV membrane in the submacular region (*arrows*). There was an increase in both IBA1^+^ (**C**) and HLA-DR^+^ macrophages (**D**) associated with the CNV in neovascular AMD eyes. (**A**) Merged channels; (**B**) desaturated UEA channel; (**C**) IBA1 channel; (**D**) HLA-DR channel. *Scale bar*: 1 mm.

**Figure 10:**
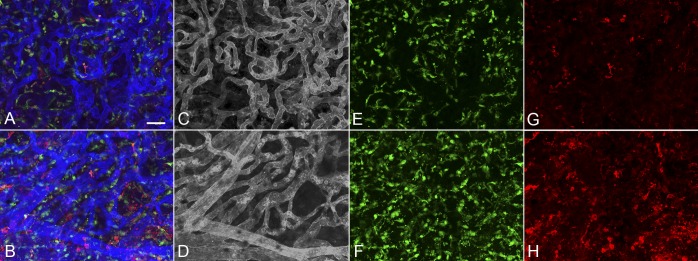
Higher magnification images of the paramacular choroid adjacent to CNV (**A**, **C**, **E**, **G**) and submacular field of choroid (**B**, **D**, **F**, **H**) in the neovascular AMD demonstrate attenuated choriocapillaris in advance of CNV (**C**) and a nonlobular pattern of CNV in submacular region (**D**). Very few IBA1- or HLA-DR^+^-labeled macrophages were ramified in paramacular choroid (**E**, **G**) or within the CNV membrane (**F**, **H**). There were significantly more HLA-DR^+^ macrophages in the CNV than in the non-CNV region. (**B**) Merged channels; (**C**, **D**) desaturated UEA channel; (**E**, **F**) IBA1 channel; (**G**, **H**) HLA-DR channel. *Scale bar:* 25 μm.

In eyes with neovascular AMD, large florid CNV formations were present in the submacular choroid, and numerous macrophages were associated with these formations (Fig. 9). There was attenuation of the CC adjacent to the CNV formations, as has been observed previously.^26^ In nearly all cases, only macrophages associated with the CNV formation could be analyzed, as the CNV firmly adhered to choroid and, therefore, it obscured the underlying choroid. Although there were more IBA1^+^ cells present in CNV, counts were not significantly higher than those in controls, except in nonmacula; however, there were significantly more HLA-DR^+^ macrophages associated with CNV (*P* = 0.001) (Fig. 2). Submacular macrophages exhibited an amoeboid morphology with few to no processes in eyes with neovascular AMD (Figs. 3, 10). These macrophages were significantly smaller (*P* < 0.00001) (Fig. 4A) and more round (*P* < 0.00001) (Fig. 4B) than those in controls.

A comparison of early and intermediate AMD, GA, and neovascular AMD eyes with ANOVA analysis revealed significant differences in macrophage volume (*P* < 0.0001). Mean macrophage volume was significantly higher in neovascular AMD (1,389.4 μm^3^) than in GA and early/intermediate AMD (1986.7 μm^3^ and 2180.0 μm^3^, respectively). No significant differences in macrophage sphericity, IBA^+^ cell count, or HLA-DR^+^ macrophage count were observed in these comparisons of AMD groups.

## Discussion

Macrophages are resident cells of the normal choroid. Under physiological conditions they perform a housekeeping role, removing debris and dead and dying cells in the normal process of cell turnover. Although a role for these macrophages in AMD was suggested nearly 30 years ago,^27^ our study is the first to quantify the number of macrophages (IBA1^+^) and to quantify macrophage activation markers like HLA-DR in aged control and AMD human choroids. There were significantly more HLA-DR–expressing macrophages in the submacular choroid in all AMD groups than in aged control eyes. A notable finding was the reduced volume and increased sphericity of these cells in AMD choroids, also indicating activation.

The present study demonstrates that, in the normal aged choroid, there are numerous tissue macrophages, of which a relatively low percentage (approximately 35% in submacula) are presumably activated (i.e., express HLA-DR). Most had a ramified morphology with several main trunks, each with fine, thin processes and a relatively large cell volume. Based on these observations, most of the cells could therefore be classified as anti-inflammatory M2 macrophages.^28^ In support of this hypothesis and using quantitative real-time polymerase chain reaction for representative M1 (CXCL11) and M2 (CCL22) transcripts, Cao et al.^29^ demonstrated high M2-chemokine transcripts and a low M1-to-M2 chemokine transcript ratio in aging non-AMD eyes. M2 macrophages are thought to perform the beneficial, long-term housekeeping role. Real-time movies of the CX3CR1^GFP/+^ mouse demonstrate that these macrophage processes are constantly sampling the environment in fulfillment of their role as sentinels and housekeepers.^30^ The study also demonstrated that these dendritiform myeloid cells were perivascular and touched the choroidal vessels,^31^ which we also observed in human choroid (Supplementary Movie S1).

The pathogenesis of early AMD is characterized by the thickening of Bruch's membrane due to lipid and protein accumulation. This leads to formation of sub-RPE deposits that occur as discrete accumulations or drusen. It is widely accepted among clinicians that the number, size, and confluency of drusen in the macula are major risk factors for both atrophic and neovascular disease. Hageman et al.^32^ hypothesized that drusen are biomarkers for immune-mediated processes at the Bruch's membrane/RPE border, and Buschini et al.^33^ hypothesized that debris accumulation may exceed the capacity for clearance by microglia/macrophages, resulting in chronic local inflammation. Histopathologic studies have shown macrophages localized to pathologic areas of Bruch's membrane in early AMD where they insert processes into Bruch's membrane deposits, presumably to scavenge debris.^32^ These deposits contain many molecules that can activate macrophages^34^ including C3a and C5a.^35^ Resident choroidal macrophages in normal human eyes do not express inducible nitric oxide synthase (iNOS), but the presence of soft drusen or continuous thick basal laminar deposits is associated with macrophage recruitment to Bruch's membrane and iNOS expression (a characteristic of M1 macrophages).^16^ This suggests an altered resident choroidal macrophage phenotype and local immunomodulatory differences between macrophages in Bruch's membrane and the choroid. In addition, the pro-inflammatory molecule C-reactive protein can also activate macrophages and is elevated in both AMD choroid^36^ and serum.^7^ If macrophages convert to M1 in the AMD choroid after encountering these proinflammatory molecules, they can incite and exacerbate the inflammatory responses to injury.

In early AMD, there was a significantly higher number (IBA1^+^) and activation (HLA-DR^+^) of submacular choroid macrophages. Perhaps the most significant change we observed in early AMD was a reduction in macrophage volume and increase in sphericity. Microglia and macrophage activation is usually measured by immunohistochemical assessment of the specific antigenic markers, but this does consider morphologic changes and shape descriptors. Microglia show a wide range of morphological transformations from highly ramified with thin branches to hypertrophic and amoeboid with no branching. Macrophages are less prone to morphological changes; however, a certain amount of shape change is possible as they can change their contractility state and establish interactions with both the extracellular matrix and cell surface adhesion molecules.^37^ McWhorter et al.^28^ showed in vitro that a change in macrophage shape from ramified to round indicates a change in cellular markers from M2 to M1. Studies are currently underway in our laboratory to quantify the number and distribution of M1 and M2 phenotypes in an effort to better define the choroidal macrophage populations in aged control eyes compared to eyes with AMD.

Hypoxia also has a dramatic influence on the phenotype and functions of macrophages.^38^ The CC loss associated with early AMD may make the submacular choroid and RPE hypoxic. Hypoxia can activate macrophages, causing them to express iNOS (a marker for M1 macrophages) and activate NF-κB, creating a pro-inflammatory environment.^39^ The hypoxic microenvironment imposes a metabolic adaptation to macrophages, skewing their functions towards a mitogenic, pro-invasive, and also pro-angiogenic phenotype. It is possible that the changes in macrophage volume and shape observed in early AMD may represent an early phenotypic response to choroidal hypoxia. Another suggestion of hypoxia in early and late AMD choroid is expression of HLA-DR by choriocapillaris endothelial cells (results not shown), which, as demonstrated by Lahat et al.,^40^ occurred in hypoxic endothelial cells in vitro.

In GA eyes, there were significantly more activated macrophages in the area of submacular choroid associated with RPE atrophy compared to control eyes. It is well known from clinical and histopathologic studies that choroidal thickness in GA can be less than half of the choroidal thickness of aged control eyes. As a result, our macrophage counts could be significantly greater in GA eyes if the numbers were expressed as cubic millimeters of tissue rather than square millimeters. The morphology of submacular macrophages in GA eyes had amoeboid morphology with few to no branches or processes, similar to what we observed in neovascular AMD. Although there were no differences in numbers of cells in the atrophic, border, and nonatrophic regions, macrophages were more ramified in border and nonatrophic regions.

In eyes with nAMD, there was a significant increase in activated HLA-DR^+^ macrophages associated with CNV. As we were unable to visualize intrachoroidal cells underlying these lesions in most eyes, as a result of the inability to excise the CNV membrane without damaging the underlying tissue, our numbers are likely an underestimate of the true number of macrophages in the region. Only cells in the CNV formations were counted. In one eye where the CNV was loosely adhered to the submacular choroid and we were able to remove the membrane, we counted a similar number of activated macrophages in the choroidal stroma underlying the lesion as in the CNV itself (data not shown). Most macrophages associated with CNV (and within the choroidal stroma) had amoeboid morphology with few to no branches. Whether these were blood-derived macrophages or resident cells is unclear from our analysis.

Numerous histopathologic studies have observed macrophages in CNV membranes in AMD, and approximately 60% of surgically excised CNV membranes contained macrophages in one study.^41^ The causal role of macrophages in regulating severity of CNV has been shown in a mouse model of experimental CNV, in which macrophage depletion decreased CNV size by approximately 50%.^42^ Approximately 90% of the macrophages in laser-induced experimental CNV are blood-derived.^43^ It therefore seems reasonable to assume that some of the HLA-DR^+^ cells associated with CNV in the human specimens evaluated herein were blood-derived.

This study quantified the number of macrophages in human choroids for the first time and demonstrates that the number of activated macrophages (HLA-DR^+^) is significantly elevated in submacular choroid in all AMD groups compared to controls. However, these numbers likely represent an underestimate of the true cell count, as they were only quantified in the flat perspective and the counts were expressed in square millimeters. Macrophages also have a lower volume and higher sphericity among all AMD groups than in controls, also indicative of activation. Skeie and Mullins^44^ recently questioned whether macrophages in AMD were “friend or foe.” The significant increase in their number in early AMD may suggest that they are a friend in cleaning debris from RPE/Bruch's membrane.^33^ They were also significantly activated in early/intermediate AMD eyes, suggesting that macrophage activation could play a causal role in progression to AMD pathology. Perhaps the debris they scavenge may be responsible for their activation. The significantly higher number of HLA-DR^+^ cells observed in CNV could suggest that they are contributing angiogenic factors to fuel the CNV, or conversely, inducing or participating in its regression. Macrophage activation in AMD demonstrated by our study supports the concept that AMD is an inflammatory disease; however, preventing activation of macrophages could result in reduced removal of debris in AMD, prolonging the presence of this toxic debris in and on Bruch's membrane. Timing of efforts to control macrophage activation, therefore, must be carefully chosen so that they can fulfill their natural role in surveying their milieu and removing debris and dead cells that result from aging.

## Supplementary Material

Supplement 1

Supplement 2

Supplement 3
