# Supplementary material for: Distribution and Quantification of Choroidal Macrophages in Human Eyes With Age-Related Macular Degeneration
Source: Invest Ophthalmol Vis Sci. 2016 Nov;57(14):5843–55. doi: 10.1167/iovs.16-20049 (PMC5098452; doi:10.1167/iovs.16-20049)
Supplement: Supplement 3 [file iovs-57-11-45_s03.pdf]

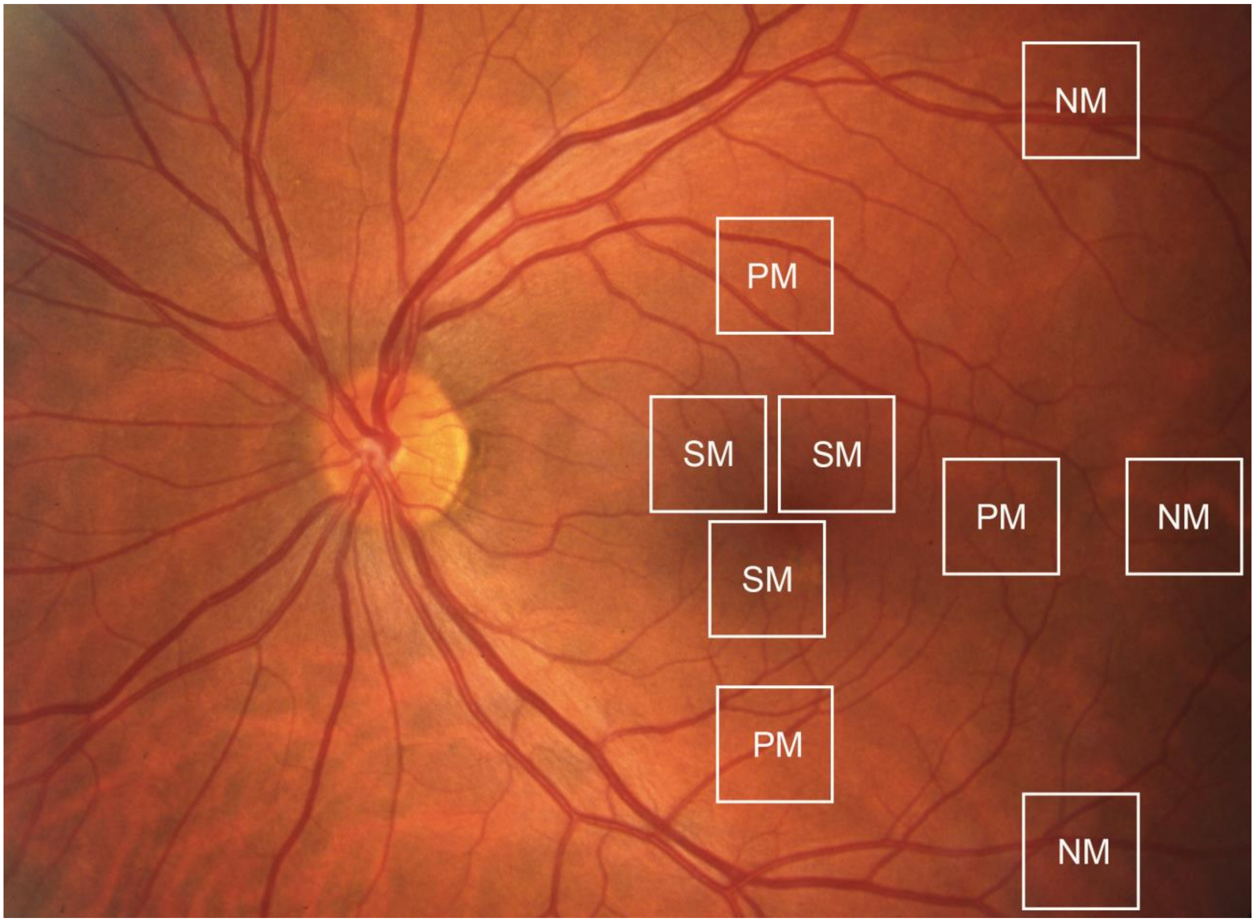

Supplemental Figure 1

Fundus photo showing regions of choroid where macrophage counts were made. (SM = submacular; PM = paramacular; NM = nonmacular)

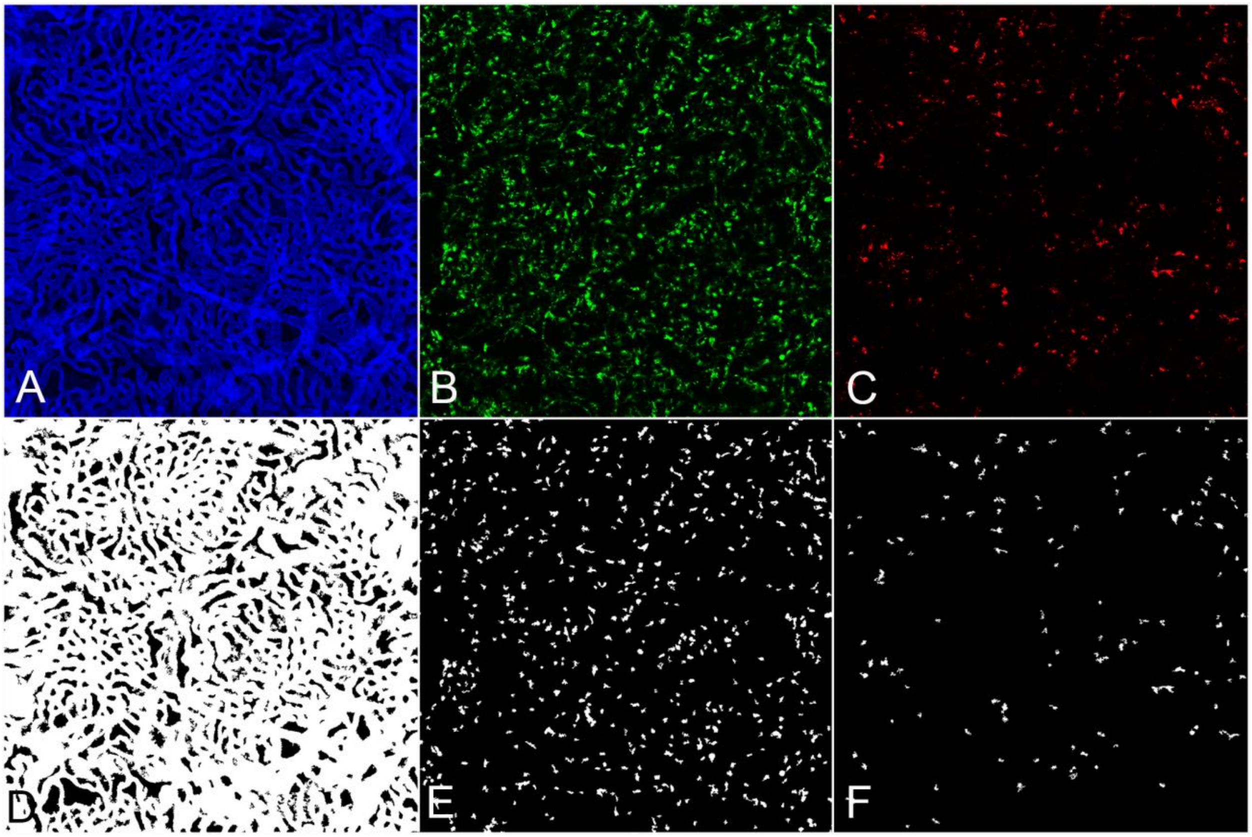

Supplemental Figure 2

Blue (UEA), green (IBA1) and red (HLA-DR) channels in a 1X1mm image of a submacular field in an aged control choroid before processing (A-C) and following thresholding and conversion to binary for image analysis (D-F).

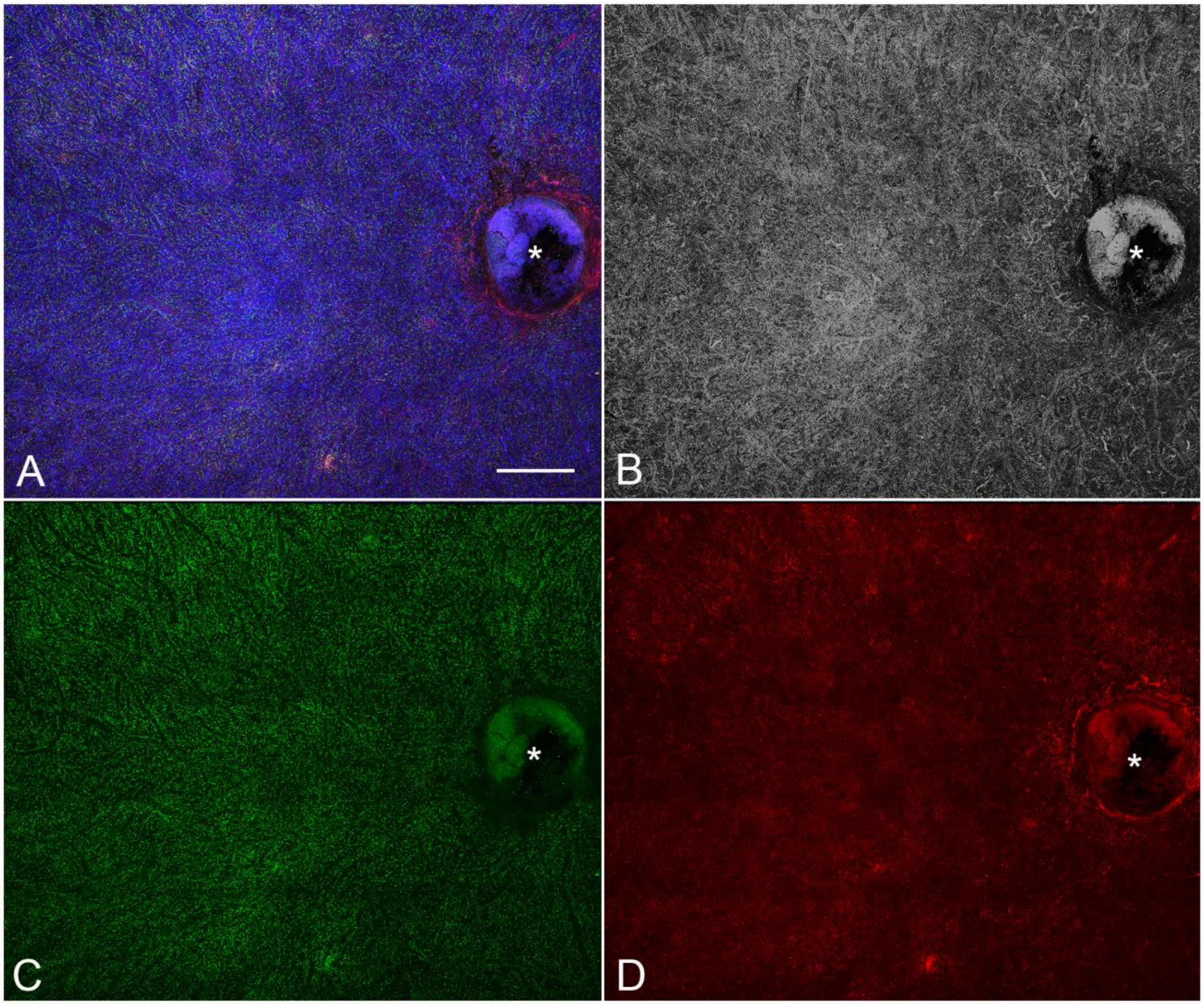

Supplemental Figure 3

Low magnification image of the posterior pole region of choroid from an aged control subject (Subject #1) showing homogeneous distribution of UEA^+ vasculature, IBA1 labeled and HLA-DR^+ macrophages. (A = merged channels; B = desaturated UEA channel; C = IBA1 channel; D = HLA-DR channel; *= optic nerve; Bar = 1mm)

Supplemental Movie 1

A dimensional volume rendering of a control choroid from a 73 year-old Caucasian female. The choroidal vasculature is stained with UEA lectin (red) and macrophages

labeled for IBA1 (green). An intermediate sized vessel in Sattler’s layer traverses this area under choriocapillaris.

Supplemental Movie 2

A dimensional volume rendering of a geographic atrophy choroid from a 92 year-old Caucasian male. The highly attenuated choriocapillaris is stained with UEA lectin (red) and macrophages labeled for IBA1 (green).
